# Hybrid coordination for the fast formation building of multi-small-AUV systems with the on-board cameras and limited communication

**DOI:** 10.7717/peerj-cs.1358

**Published:** 2023-04-24

**Authors:** Xiaomin Wang, Xiaohan Zhang, Zhou Zheng, Xu Kong

**Affiliations:** 1 College of Electrical Engineering and Automation, Shandong University of Science and Technology, Qingdao, China; 2 Shandong Provincial Academy of Educational Recruitment and Examination, Jinan, China

**Keywords:** Multi-small-AUV system, Pattern formation, Visual servoing, Fast convergence, Hybrid coordination strategy

## Abstract

Formation building for multi-small-AUV systems with on-board cameras is crucial under the limited communication underwater environment. A hybrid coordination strategy is proposed for the rapid convergence to a leader-follower pattern. The strategy consists of two parts: a time-optimal local-position-based controller (TOLC) and a distributed asynchronous discrete weighted consensus controller (ADWCC). The TOLC controller is designed to optimize the assignation of AUVs’ destinations in the given pattern and guide each AUV to its destination by the shortest feasible distance. The ADWCC controller is developed to direct the AUVs blocked by obstacles to reach their destinations with the information from the perceived neighbors by on-board cameras. The rapidity of the proposed strategy is theoretically discussed. The effectiveness of the proposed algorithm has been verified in the simulation environments in both MATLAB and Blender.

## Introduction

The reliable underwater missions to search small objects and to sample colorful bio-information are important yet challenging in the communication-limited unknown underwater environment (Lu et al., 2019). The high-effectivity multi-small-AUV system (MSAS) navigated by low-cost and low-power consumption on-board colorful cameras provides a solution to complete these tasks in the water (Nash et al., 2021; Berlinger, Gauci & Nagpal, 2021a; Wang et al., 2020). The MSASs extend the working range of on-board cameras, while the on-board cameras capture the colorful information and perform partial implicit communications with each other.

Coordinated control is important for AUVs to accomplish tasks cooperatively, including building a pattern to bring AUVs from disorder to order, maintaining the pattern while performing the mission, and rebuilding the pattern when the system is obstructed by obstacles. There have been a great number of control methods, which are categorized into position-, displacement- and distance-based methods according to the sensors perceiving environments (Oh, Park & Ahn, 2015). The position-based method needs global sensors to provide global positions, but it is fast with little dependence on the topological relationship among robots, so that it is usually used in the empty outdoor (Yu & Barca, 2015). The displacement- and distance-based methods achieve the coordination control with the local information sensed by local sensors, but they need the topological relathionship as the control constraints, and they are usually used in the local or communication-limited environments (Wang et al., 2017; Yun & Doik, 2021).

Advances in computer vision and robotics have led to increasing research on local control (displacement- and distance-based control) with visual servoing in communication-constrained environments (Montijano et al., 2016; Rioux et al., 2017; Lobos-Tsunekawa, Leiva & del Solar, 2018; Manzanilla et al., 2019). Most are researching the vision-based land and air robotic systems (Lin et al., 2020; Zhou et al., 2022). Recently, there have been growing appeals for the formation control of vision-based MSASs, such as the self-organization control strategy for imitating the dynamic circle and milling formations of fish schooling (Berlinger, Gauci & Nagpal, 2021a) and the vision-based escape strategy for evading the predators of fish schooling (Berlinger, Wulkop & Nagpal, 2021b).

In general, formation control with on-board cameras is a distributed consensus problem. Many distributed control theoretical results have been obtained for multi-robot systems based on local information, including linear or non-linear, continuous or discrete, low-order or high-order consensus algorithms with undirected or directed, time-invariant or time-variant topologies (Yu et al., 2020; Duan & Chow, 2019; Wang & Su, 2019; Mu et al., 2019). Now there are also increasing number of studies on the distributed control of multi-AUV sysetems with local information. To solve the problem of the uncertain parameter in the kinetic model, the sliding controllers based on backstepping control are studied (Sun et al., 2018; Li, Yuan & Zhang, 2019). Due to the real-time constraints in practice, a series of finite-time controllers are proposed to achieve formation control (Fan & Zhao, 2021; Chen et al., 2021). Meantime, considering the limited communication in the water, the time delay or/and jointly connected interactive topology are considered in the formation control (Yan et al., 2022a; Zhang, Zhou & Yao, 2023). Further, some researchers introduce deep learning into the non-linear multi-AUV systems to optimize the uncertain parameters (Yuan, Licht & He, 2017; Thuyen, Thanh & Anh, 2023). Most of the above works of literature study the continuous control of multi-AUV systems, but the visual perception system provides discrete information in this study.

There are a few studies on the discrete control of multi-AUV systems. Zhang et al. (2021) studies the problem of formation maintenance of the discrete-time leader-follower multi-AUV system in which the communication topology is a time-varying delay-directed graph. Yan et al. (2022b) studies the trajectory tracking controllers under the leader-follower and virtual leader structures respectively in discrete systems under weak communication conditions and discusses their convergence. The literature Zeng et al. (2022) provides a finite-time discrete coordinated formation controller for the multi-AUV systems which have input saturation constraints, time-varying communication delays, and variable weighted topologies. Besides, an increasing number of studies are on the multi-robot system with on-board vision feedback. Literature He et al. (2022) studies a formation tracking control problem of multiple nonholonomic autonomous vehicles with modeling uncertainty and the limited sensing capability of on-board vision. Literature Miao et al. (2021) proposes a Nussbaum gain adaptive controller and a static nonlinear gain controller for the characteristics of visual servoing to complete the leader-follower trajectory tracking control problem. However, there are only a few studies on the formation control of discrete multi-AUV systems by virtue of feedback from onboard vision systems (Wang et al., 2020). And most of them do not take the time consumption problem into account. In fact, the complexity of underwater environment brings challenges to the endurance of AUVs in the reality. Fast convergence to the given pattern is beneficial to save energy and complete the tasks. The time complexity of algorithmes and the movement distance of each AUV are two aspects to reduce the energy consumption.

Inspired by the optimization algorithms (Wang et al., 2022), the purpose of this article is to report a strategy to fast build a given pattern depending on the perception of on-board cameras. Based on the position-based method, we construct a common frame with the shared neighbors’ information and get the initial distribution. Then we propose a time-optimal local-position-based controller (TOLC) to determine the pattern distribution and generate an optimal initial trajectory with a short distance for each AUV. At the same time, we have to realize that the main sensors are on-board cameras so that each AUV has no ability to update its position in the common frame. Consequently, TOLC is invalid when the AUVs are blocked by obstacles. In this case, the distributed consensus algorithm is introduced to update trajectories based on the perceived neighbors. Combined with the abundant information sensed by cameras and the expected position relationship (EPR) obtained by TOLC, an asynchronous discrete weighted consensus controller (ADWCC) is proposed to update the trajectories with fast convergence for the hampered AUVs.

Our main contributions are summarized in four points: (1) A fast hybrid coordination including a TOLC and an ADWCC is proposed for MSASs to achieve the formation building quickly by the navigation of on-board cameras. (2) The TOLC algorithm, from a geometric point of view, is analyzed and proposed to approach the expected formation to the initial distribution and generate non-intercrossing short initial trajectory, to reduce the energy consumption of the robot. (3) The ADWCC algorithm in view of local neighbor information from cameras is proposed to update the trajectories for AUVs hampered by obstacles to avoid obstacles and complete formation construction, and a weight calculation method based on spectral radius constraint is given to accelerate the convergent rate of each updating loop. (4) A theoretically discussion about the rapidity of the hybrid coordination is given from the perspective of the matrix.

## Problem description

**Figure 1:**
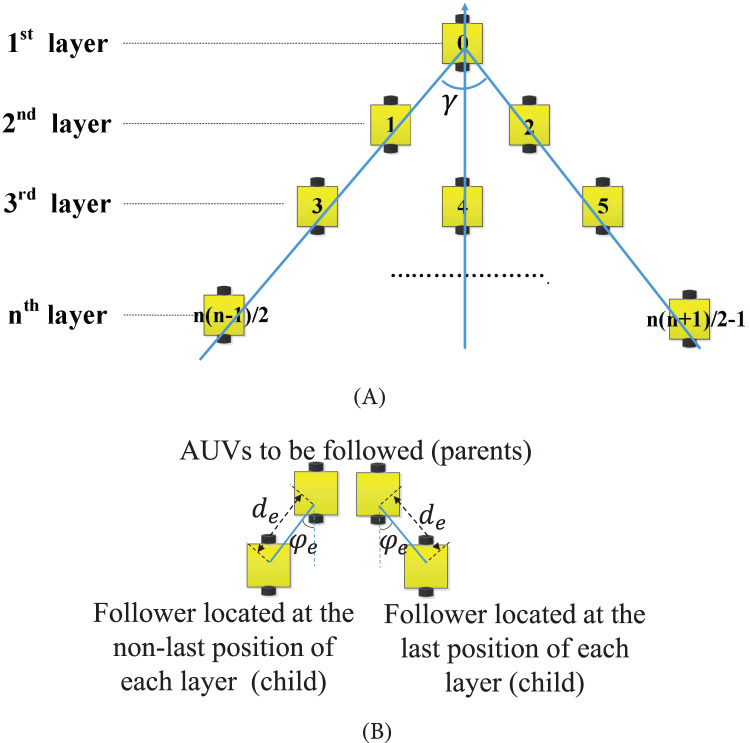
Planar pyramid pattern. (A) The sketch of the planar pyramid pattern. (B) The location diagram between followers (children) and the followed robots (parents).

The formation building of MSASs needs to be fast with low time and energy consumption for saving more energy to accomplish the tasks, even in the obstacle environment. Due to the attenuation of light, the camera in the water has a limited view angle and short depth of view. Consequently, we introduce the planar pyramid pattern with leader-follower structure, which is suitable for the camera perception characteristics, shown in Fig. 1. In the planar pyramid pattern, each robot to be followed is denoted as a parent, while its followers are denoted as its children. Each robot has only one parent. For most robots, their parents are located at their right-front. But for the robots located at the right end of each layer, their parents are designated at their left-front, since there are no robots in their right-front. Their relative distances and relative angles are fixed, noted by ${d_e}$ and ${\varphi _e}$. The perception topologies of MSASs can be expressed by directed graphs (digraph). Each AUV is a node, while the perceived neighbors are connected by a directed channel since the visual perception is unidirected. For example, if the on-board camera of AUV- $i$ finds AUV- $j$, the channel from AUV- $i$ to AUV- $j$ is noted by ${a_{ij}} \gt 0$, othervise ${a_{ij}} = 0$.

$Assumption\;1$: For a MSAS with *N* AUVs, noted by $S = \{ {R_1}, \ldots ,{R_N}\}$, each AUV- $i$ finds at least one neighbor, noted by a sub-set ${S_i} = \{ {R_m}, \ldots ,{R_q}\}$. The topologies of MSASs are strongly connected digraphs, noted by $\bigcup\nolimits_{i = 1}^N {{S_i}} = S$. When ${a_{ij}} \gt 0$, then ${a_{ji}} \gt 0$, but usually ${a_{ij}} \ne {a_{ji}}$ for designing weights of each AUV. Where, ${R_m}$, ${R_q}$, ${R_1}$, ${R_N}$ are the notes of AUV- $m$, AUV- $q$, AUV- $1$, AUV-*N*.

According to the connectivity assumption in Assumption 1, we construct the common frame ${o_c}{x_c}{y_c}$ with shared informaiton and the transformation strategy in Wang et al. (2019), and obtain the initial distribution ${{\bf{p}}_c} = \{ {{\bf{p}}_{c{R_i}}}|{R_i} \in S\}$ in the ${o_c}{x_c}{y_c}$ (Wang et al., 2019).

With the obtained initial distribution ${{\bf{p}}_c}$, to complete the formation building quickly, the moving distance of each AUV is supposed to be the shortest. Then the pyramid distribution to be determined, ${{\bf{p}}_p} = \{ {{\bf{p}}_{c{R_i}}}|{R_i} \in S\}$, is supposed to tend to the initial distribution for ensuring the largest overlapped area enclosed by the two distributions. Meanwhile, the sum of the distances between the assigned destinations in the pyramid distribution and the initial positions is the shortest to obtain the EPR between the positions in initial and pyramid distributions, shown in (1).

(1) $$\left\{ {\eqalign{ {{{\bf{p}}_p} = {\rm{argmin}}(\Omega ({{\bf{p}}_c})\bigcup \Omega ({{\bf{p}}_p}))}\quad \quad\quad\quad \cr \!\!\!\!\!\!\!\!\!{{F_{EPR}} = {\rm{argmin}}(\sum\nolimits_{{R_i} \in S} d is({{\bf{p}}_{c{R_i}}},{{\bf{p}}_{p{R_i}}}))} \cr } } \right.\!\!\!\!\!\!,$$

where $\Omega ({{\bf{p}}_c})$ and $\Omega ({{\bf{p}}_p})$ are the areas enclosed by initial and pyramid distributions, respectively; and ${F_{EPR}} = \{ ({{\bf{p}}_{c{R_i}}},{{\bf{p}}_{p{R_i}}})|{R_i} \in S\}$ stores the optimized assignment results of the positions in both distributions.

However, the time complexity of the optimal algorithms is usually high, even there is no optimal solution. Consequently, to simplify the algorithm as well as to shorten the moving distance of AUVs, we give a compromise and propose a lower complexity algorithm that can generate the sub-optimal pyramid distribution and the sub-optimal assignment of positions, shown in (2).

(2) $$\left\{ {\eqalign{ {{{\widehat {\bf{p}}}_p} = {\rm{SMALL}}(\Omega ({{\bf{p}}_c})\bigcup \Omega ({{\widehat {\bf{p}}}_p}))} \quad\quad\qquad \cr \!\!\!\!\!\!\!\!{{{\hat F}_{EPR}} = {\rm{SMALL}}(\sum\nolimits_{{R_i} \in S} d is({{\bf{p}}_{c{R_i}}},{{\widehat {\bf{p}}}_{p{R_i}}}))} \cr } } \right.\!\!\!\!\!\!,$$

where ${\rm{SMALL}}(f(x)) \in {\rm{U}}({\rm{argmin}}(f(x))$, ${\rm{U}}({\rm{argmin}}(f(x))$ is the neighborhood of ${\rm{argmin}}(f(x))$, it means that there is a $x$ to make the value of $f(x)$ small but not the minimum value, just in the ${\rm{U}}({\rm{argmin}}(f(x))$; ${\widehat {\bf{p}}_{p{R_i}}}$ is one position in the sub-optimal pyramid distribution ${\widehat {\bf{p}}_p}$, ${\hat F_{EPR}} = \{ ({{\bf{p}}_{c{R_i}}},{\widehat {\bf{p}}_{p{R_i}}})|{R_i} \in S\}$ stores the sub-optimal assignment results of the positions in both distributions.

As a result, the lines connecting the assigned positions in ${\hat F_{EPR}}$ become the initial trajectories for AUVs. However, when one or more AUVs are hampered by obstacles, these hampered AUVs hardly re-obtain their positions in the common frame due to the local perception of on-board cameras. In this case, the distributed control methods are needed to continuously update the trajectories loop by loop (“search, compute, move”) for the hampered AUVs with the perceived neighbors until the formation is completed. Limited by the short working range of cameras, each AUV needs time to perceive the surroundings. Coupled with the dynamics of the robot, the perceived neighbors are time-variant. Then the trajectory updating is a discrete consensus problem with time-variant topologies. To accelerate the convergence speed, a trajectory updating controller meeting (3) is needed to generate the fastest convergent trajectory in each updating loop.

(3) $$\left\{ {\matrix{ {{\bf{u}}[{{(k + 1)}_i}] = {\bf{W}}({G_i}[{k_i}]){{\bf{e}}_i}[{k_i}] + {\bf{W^\prime}}} {({G_i}[{k_i}]){{\bf{e}}_{io}}[{k_i}]} \quad \qquad\cr {{\bf{W}}({G_i}[{k_i}]) = f({\rm{argmin}}\{ (\rho ({{\bf{M}}_j}({G_i}[{k_i}])|j \in [1, \ldots ,{N_{{G_i}}}])\} )} & {} \cr } } \right.,$$

where ${\bf{W}}({G_i}[{k_i}])$ and ${\bf{W^\prime}}({G_i}[{k_i}])$ are the unknown time-variant matrix, ${{\bf{M}}_j}({G_i}[{k_i}])$ is one matrix to express the sub-digraph ${G_i}[{k_i}]$ composed by AUV– $i$ and its perceived neighbors in the ${k_i}$ updating loop, ${N_{{G_i}}}$ is the number of matrix to express the sub-digraph ${G_i}[{k_i}]$. ${{\bf{e}}_i}[{k_i}]$ and ${{\bf{e}}_{io}}[{k_i}]$ are the relative position error matrices of the found neighbors and obstacles in the ${k_i}$ loop.

$Problem\;1$: For a MSAS with an on-board camera as the main sensor for each AUV and the time-variant connected digraph as its topology, find a controller to determine the sub-optimal pyramid distribution, assignment results meeting (2) and generate the initial trajectories, and find a controller in (3) to make AUVs update their trajectories to bypass obstacles and fast move to the destination to let AUVs achieve the formaiton building.

## Methods

This section describes the proposed hybrid coordination to solve Problem 1. To achieve (2), a time-optimal local-position-based controller (TOLC) is put forward, while to update trajectories and converge to the destination quickly, an asynchronous discrete weighted consensus controller (ADWCC) is reported.

### Time-optimal local-position-based controller

To propose TOLC for generating the short and non-intercrossing trajectories, we introduce lemma 1 and lemma 2.

$Lemma\;1$ (Horn & Johnson, 2012): ${{\bf{C}}_x}$ and ${{\bf{p}}_{ave}}$ are the covariance matrix of the positions and the average position in a distribution ${{\bf{p}}_\Omega }$, respectively. Then ${{\bf{C}}_x}$ can describe the distribution ${{\bf{p}}_\Omega }$, the eigenvectors ${{\bf{v}}_i}$ and eigenvalues ${\lambda _i}$ of ${{\bf{C}}_x}$ can represent the rotation and the scale of ${{\bf{p}}_\Omega }$, respectively, while ${{\bf{p}}_{ave}}$ can decide the position of ${{\bf{p}}_\Omega }$.

**Figure 2:**
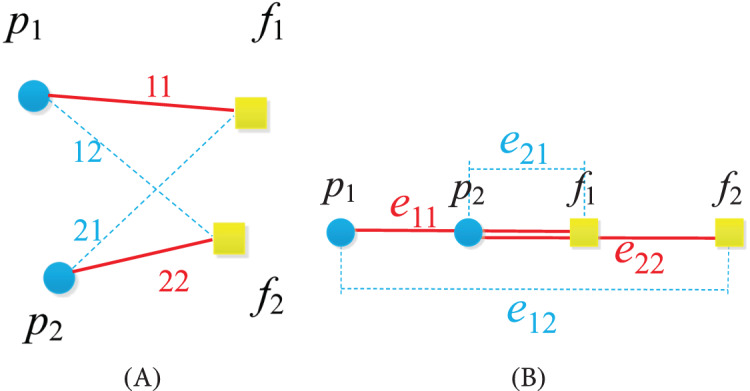
The illustration of lemma 2. (A) The general situation. (B) The overlapping situation.

$Lemma\;2$ (Fujinaga et al., 2015): Let ${p_1}$, ${p_2}$ are the two distinct positions in the initial distribution, while ${f_1}$, ${f_2}$ are two distinct positions in the pyramid pattern, shown in Fig. 2. Then ${e_{11}}$ and ${e_{22}}$ are never cross, even though they are overlapping (shown in Fig. 2B), if ${d_{{e_{11}}}} + {d_{{e_{22}}}}\lt{d_{{e_{12}}}} + {d_{{e_{21}}}}$ and $max({d_{{e_{11}}}},{d_{{e_{22}}}})\lt max({d_{{e_{12}}}},{d_{{e_{21}}}})$.

$Remark\;1$: The overlapping situation is that all the points are alligned. In this case, ${d_{{e_{11}}}} + {d_{{e_{22}}}} = {d_{{e_{12}}}} + {d_{{e_{21}}}}$, but $max({d_{{e_{11}}}},{d_{{e_{22}}}})\lt max({d_{{e_{12}}}},{d_{{e_{21}}}})$, shown in Fig. 2B, then two robots do not collide with each other, we say that ${e_{11}}$ and ${e_{22}}$ are not inter-crossing, noted as ${e_{11}} \cap {e_{22}} = \emptyset$.

To meet the requirement of (2), the error norm of covariance matrices of both distributions needs to be small according to lemma 1. With lemma 2, the edges connecting the assignment results are not inter-crossing. The approximate loss function is expressed by (4).

(4) $$\left\{ {\matrix{ {{\widehat {\bf{p}}}_p}={\rm SMALL}(||Cov({\bf p}_c)-Cov({\bf p}_p) ||) \qquad \qquad \quad \qquad\cr \!{\hat{F}_{EPR}}=\{e_1\cap e_2 \ldots\cap e_N= { / \hskip-6pt O\!}\; |e_i=p_{cR_{i}p_{p}R_{i}},i\in[1, \ldots, N] \} \cr } } \right.,$$

where $Cov({{\bf{p}}_c})$ and $Cov({{\bf{p}}_p})$ are the covariant matrices of initial distribution ${{\bf{p}}_c}$ and pyramid distribution ${{\bf{p}}_p}$, respectively, ${p_{c{R_i}}}$ and ${p_{p{R_i}}}$ are two end points of the edge ${e_i}$ connecting the assignment pair ${{\bf{p}}_{c{R_i}}}$ and ${{\bf{p}}_{p{R_i}}}$.

Then TOLC is proposed to minimize the values of cost function. The controller includes two parts: a pattern determination strategy to determine the pyramid distribution, and an assignment strategy to assign the positions in both distributions.

#### Pattern determination strategy

To make ${{\bf{p}}_p}$ approximate ${{\bf{p}}_c}$, we propose a pattern determination strategy in view of a geometric way to reduce the amount of calculation. This strategy is expressed by (5).

(5) $$\left\{ {\matrix{\!\!\!\! {{{\bf{o}}_p} = {1 \over N}\sum\nolimits_{i = 1}^N {{{\bf{p}}_{c{R_i}}}} } \cr {{{\bf{v}}_{pmax}} = {{\bf{v}}_{cmax}}} \qquad \cr } } \right.,$$

where ${{\bf{o}}_p}$ is the average position of the pyramid distribution, it coincides with the average position of the initial distribution, ${{\bf{v}}_{pmax}}$ and ${{\bf{v}}_{cmax}}$ are the associated eigenvectors of the largest eigenvalues of $Cov({{\bf{p}}_c})$ and $Cov({{\bf{p}}_p})$, respectively.

**Figure 3:**
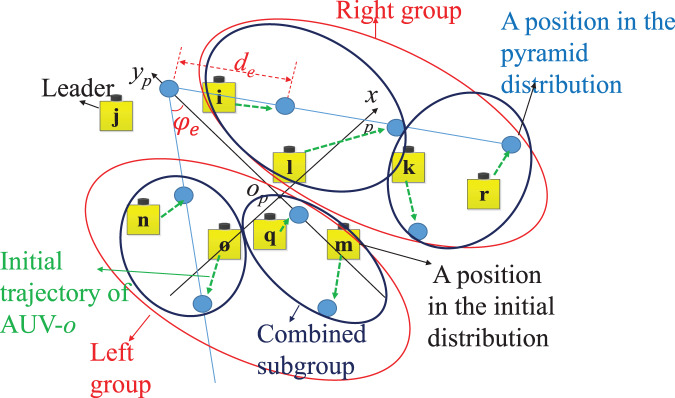
The explanation of the assignment strategy.

Consequently, the pyramid frame, ${o_p}{x_p}{y_p}$, is constructed as shown in Fig. 3, where ${{\bf{o}}_p}$ is the origin point of the pyramid frame, ${{\bf{v}}_{pmax}}$ is the $y$ axis, $x$ axis is perpendicular with $y$ axis in the plane. Then the positions of pyramid pattern ${{\bf{p}}_p}$ are calculated according to the definition of pyramid pattern (Wang et al., 2019).

#### Assignment strategy

Since the time complexity of an algebric method to calculate ${\hat F_{EPR}}$ is $O(n!)$, the time consumption increases dramaly when there are more robots. Herein, we design an assignment strategy, which is a combination of a geometric way and an algebric way with the time complexity $O(nlog(n))$. $O(nlog(n))$ is decided by the sorting algorithm of positions. The geometric way is employed to seperate the positions in both distributions into two groups: left group and right group. In each group, the positions of both distributions are continously seperated into subgroups with only one or two positions. The sub-groups of both distribution located at the same areas are combined together into combined sub-groups, described in Fig. 3. Subsequently, the algebric way is used to calculate the shortest distance based on lemma 2. The detail of the assignment strategy is presented below:

(1) The positions in ${{\bf{p}}_c}$ are transformed into the pyramid frame, noted by ${{\bf{p}}_{cp}}$.

(2) In the pyramid frame, ${{\bf{p}}_{cp}}$ and ${{\bf{p}}_p}$ both are evenly seperated into two groups (left group and right group) by the y axis of pyramid frame, respectively.

(3) In each group of both distributions, the positions are sorted from top to bottom and from left to right, then they are continuously devided into subgroups only including one or two positions, noted as $Sg_{ini}^i$ and $Sg_{py}^i$.

(4) The subgroups in both distributions located at the same area are combined together to a combined-subgroup, $Sg_{com}^i = Sg_{ini}^i \cup Sg_{py}^i$ (see Fig. 3).

(5) In each combined-subgroup, lemma 2 is introduced to match up the positions in both distributions. The match-up pairs ${\hat F_{EPR}} = \{ ({{\bf{p}}_{cp{R_i}}},{{\bf{p}}_{p{R_i}}})|{R_i} \in S\}$ are the assignment results, we also get the EPR, expressed by $\{ ({R_i},or{d_{{R_i}}})|{R_i} \in S\}$, where ${{\bf{p}}_{cp{R_i}}}$ is one position of ${{\bf{p}}_{cp}}$, $or{d_{{R_i}}}$ is the order of the assignment postion of ${R_i}$ in the pyramid pattern.

(6) The edges connecting ${{\bf{p}}_{cp{R_i}}}$ and ${{\bf{p}}_{p{R_i}}}$ are the initial non-intercrossing trajectories.

$Remark\;2$: The numbers of positions of both groups in each disbtribution ( ${N_L}$ and ${N_R}$) are supposed to meet $|{N_L} - {N_R}| \le 1$. If $|{N_L} - {N_R}| \gt 1$, fine-tuning is a necessary step to reallocate the positions near the boundaries of both groups until $|{N_L} - {N_R}| \le 1$. For example, the AUV- $m$ is tuned to the left group of initial distribution in Fig. 3.

Algorithm 1 is the achievement of TOLC, it determines the pyramid frame and the pyramid distribution, as well as the assignation of AUVs’ destinations in the pyramid pattern. Moreover, the edges connecting the AUVs’ initial positions and their destinations become the initial trajectories, and they are non-intercrossing based on lemma 2.

**Algorithm 1:** Time-optimal local-position-based controller.

1: **for** ${\theta _r}$ in range $[{0^\circ },{360^\circ }]$ with a step ${\theta _{step}}$: **do**
2: Each AUV perceives its surroundings at ${\theta _r}$, and extracts the neighbors’ local position ${{\bf{p}}_{ij}}$.
3: **end for**
4: All the AUVs share their neighbors’ information by communication, $\{ R_i,{N_{{R_i}}}$, $\{ {{\bf{p}}_{ij}}|j \in 1, \ldots ,{N_i}\}\}$. ( ${N_{{R_i}}}$ is the number of neighbors of AUV-*i*.)
5: ${o_c}{x_c}{y_c}$ is built by each AUV with all the neighbors’ information.
6: ${{\bf{p}}_c}$ is obtained by transforming all the positions, ${{\bf{p}}_{ij}}$, into the common frame.
7: ${{\bf{o}}_p}$ and ${{\bf{v}}_{pmax}}$ are calculated depending on ${{\bf{p}}_c}$.
8: ${o_p}{x_p}{y_p}$ is fixed according to (5), and ${{\bf{p}}_p} = \{ {{\bf{p}}_{p{R_i}}}|{R_i} \in S\}$ is identified based on the definition of pyramid pattern in Wang et al. (2017).
9: ${{\bf{p}}_c}$ is transformed into ${o_p}{x_p}{y_p}$ and noted as ${{\bf{p}}_{cp}}$.
10: ${{\bf{p}}_{cp}}$ and ${{\bf{p}}_p}$ are seperated into left group and right group by ${o_p}{y_p}$, respectively.
11: Subgroups $Sg_{ini}^i$ and $Sg_{py}^i$ are obtained by sorting the positions in each group, the combined-subgroup $Sg_{com}^i$ is also made up.
12: The ${\hat F_{EPR}}$, EPR are obtained after the distance calculation in $Sg_{com}^i$ according to lemma 2.
13: The relative distance and angle between each pair in ${\hat F_{EPR}}$ is calculated, noted as $d_{R_i}=||{\bf p}_{cpR_{i}}-{\bf p}_{pR_{i}}||$, ${\eta _{{R_i}}} = arctan \left({{{{\bf{p}}_{cp{R_i}}}[1] \;-\; {{\bf{p}}_{p{R_i}}}[1]} \over {{{\bf{p}}_{cp{R_i}}}[0] \;-\; {{\bf{p}}_{p{R_i}}}[0]}}\right)$. Then the initial non-intercrossing trajectories $\{ ({d_{{R_i}}},{\eta _{{R_i}}}),{R_i} \in S\}$ is obtained according to lemma 2.

### Asynchronous discrete weighted consensus controller

The initial non-intercrossing trajectories generated by TOLC are not updated, since TOLC is open-loop without the real-time feedback of position information. When one or more AUVs are hampered by obstacles, their motion states are cut off. In this case, the AUVs have no ability to determine their positions and further update their trajectories in the common frame. Thus, we propose the ADWCC to let the AUVs hampered update the trajectories independently and rapidly based on the local information of neighbors obtained by on-board cameras and compasses, and accomplish the formation building with low-power consumption. The time complexity is $O({n^3})$, due to the dense matrix computation. Algorithm 2 shows the process of ADWCC.

**Algorithm 2:** Asynchronous discrete weighted consensus controller.

1: AUV-*i* hampered by the obstacles switches its controller to ADWCC.
2: AUV-*i* sets its relative distance vector error ${\bf e}_i[(k+1)_i]\gt\epsilon_{tr}$ ( $\epsilon_{tr}$ is the stopping threshold).
3: **while** $||{\bf e}_i[(k+1)_i]||\gt\epsilon_{tr}$ **do**
4: **for** ${\theta _r}$ in range $[{0^\circ },{360^\circ }$] with a step ${15^\circ }$ **do**
5: AUV-*i* perceives its surroundings at ${\theta _r}$, and extracts the neighbors’ IDs and local positions ${{\bf{p}}_{ij}}$.
6: **end for**
7: AUV-*i* computes the relative distance vector error ${{\bf{e}}_i}[{(k + 1)_i}]$.
8: **if** $||{\bf e}_i[(k+1)_i]||\gt\epsilon_{tr}$ **then**
9: AUV-*i* generates the updated trajectory $({d_i}[{k_i}],{\eta _i}[{k_i}])$, where $d_i[k_i]=||{\bf e}_i[(k+1)_i]||$ and ${\eta _i}[{k_i}] = arctan\left({{{{\bf{e}}_i}[{{(k \;+\; 1)}_i}][1]} \over {{{\bf{e}}_i}[{{(k \;+\; 1)}_i}][0]}}\right)$.
10: AUV-*i* moves to a new position along $({d_i}[{k_i}],{\eta _i}[{k_i}])$.
11: **else**
12: AUV-*i* arrives at its destination and stops.
13: **end if**
14: **end while**

To achieve the formation building, the mathmatical expression of ADWCC is presented by

(6) $$\left\{
{\eqalign{
& {{{\bf{u}}_i}[{{(k + 1)}_i}] = {{\bf{e}}_i}[{{(k + 1)}_i}]}  \cr 
& {{{\bf{e}}_i}[{{(k + 1)}_i}] = {{\bf{W}}_i}[{k_i}]{{\bf{C}}_{i1}}[{k_i}]{{\bf{e}}_{id}}[{k_i}] + {{\bf{W}}_{io}}[{k_i}]{{\bf{C}}_{i2}}[{k_i}]{{\bf{e}}_{io}}[{k_i}]}
 } } \right.,$$

where ${(k + 1)_i}$, ${k_i}$ are the ${(k + 1)_{th}}$ and ${k_{th}}$ loops of AUV- $i$, respectively; ${{\bf{W}}_i}[{k_i}] = {[{w_{ij}}[{k_i}]]_{1 \times {N_i}[{k_i}]}}$ and ${{\bf{W}}_{io}}[{k_i}] = {[{w_{io}}[{k_i}]]_{1 \times {N_{io}}[{k_i}]}}$ are the weight-vectors composed by the weights of all the neighbors and all the obstacles recognized in the ${k_i}$ loop, respectively; ${w_{ij}}[{k_i}]$, ${w_{io}}[{k_i}]$ are the the weights of neighbor- $j$ and obstacle- $o$, respectively, and ${w_{io}}[{k_i}] =  1$; ${N_i}[{k_i}]$ and ${N_{io}}[{k_i}]$ are number of neighbors and obstacles, respectively; ${{\bf{C}}_{i1}}[{k_i}] = diag{({\beta _{ij}}[{k_i}])_{{N_i}[{k_i}] \times {N_i}[{k_i}]}}$ and ${{\bf{C}}_{i2}}[{k_i}] = diag{({\alpha _{ij}}[{k_i}])_{{N_i}[{k_i}] \times {N_i}[{k_i}]}}$ are two penalty matrices introduced from Bellman function to avoid the over-planning and obstacle-collision, ${\beta _{ij}}[{k_i}]$ is the factor to adjust the component of the relative distance vector error between AUV- $i$ and AUV- $j$, and ${\alpha _{ij}}[{k_i}]$ is the factor to adjust the relative distance between AUV- $i$ and obstacle- $j$, expressed by (7); ${{\bf{e}}_{id}}[{k_i}] = {[{{\bf{e}}_{ij}}[{k_i}]]_{{N_i}[{k_i}] \times 2}}$ is the matrix composed by the errors of the measured and expected distance vectors of neighbors ( ${{\bf{e}}_{ij}}[{k_i}] = {{\bf{d}}_{ij}}[{k_i}] - {\bf{d}}_{ij}^*$) in the local frame of AUV- $i$; ${{\bf{e}}_{io}}[{k_i}] = {[{{\bf{d}}_{io}}[{k_i}]]_{{N_i}[{k_i}] \times 2}}$ is the matrix composed by the measued distance vectors of obstacles ( ${{\bf{d}}_{io}}[{k_i}]$) in the local frame of AUV- $i$.

(7) $$\left\{ {\matrix{
   {{\beta _{ij}}[{k_i}] = {{|\langle {{\bf{e}}_{ij}}[{k_i}],{{\widehat {\bf{e}}}_i}[{k_i}]\rangle |} \over {|\langle {{\bf{e}}_{ij}}[{k_i}],{{\widehat {\bf{e}}}_i}[{k_i}]\rangle |\; + \;{e^{|\langle {{\bf{e}}_{ij}}[{k_i}],{{\widehat {\bf{e}}}_i}[{k_i}]\rangle | - 1}}}} \in [0,0.5]{\rm{ }}}  \cr 
   {{\alpha _{ij}}[{k_i}] = \left\{ {\matrix{
   {0,} & {{d_{io}}[{k_i}] \ge 2r}  \cr 
   {{1 \over 2}{{{d_{io}}[{k_i}]\; - \;2r} \over r},} & {{d_{io}}[{k_i}] < 2r}  \cr 

 } } \right.{\rm{  }} \in [ - 1,0]}  \cr 

 } } \right.$$

where, $d_{io}[k_i]=||{\bf{d}}_{io}[k_i]||$, $r$ is the length of the AUV.

**Figure 4:**
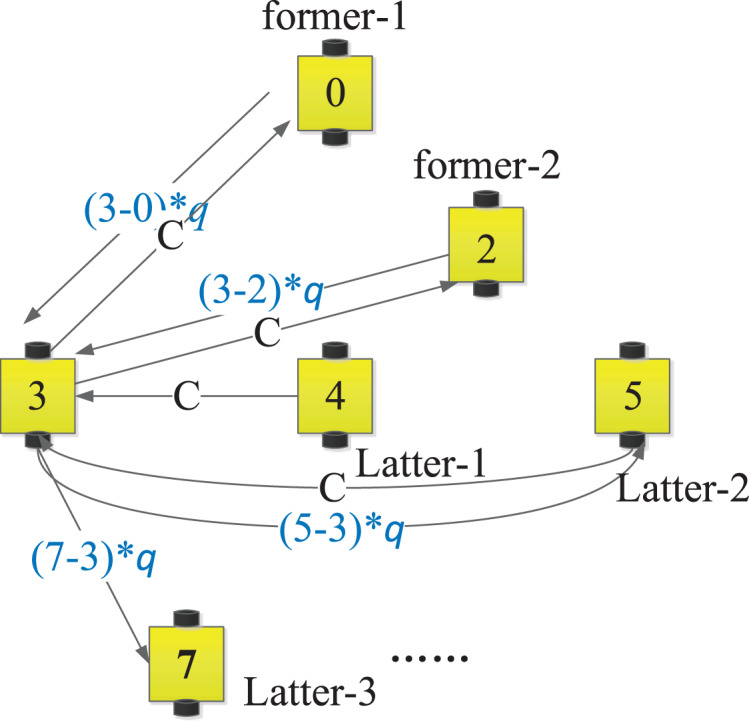
The sub-digraph ${{\bf{G}}_3}[{k_3}]$ of AUV- $3$.

To obtain ${{\bf{e}}_i}[{(k + 1)_i}]$, we report the way to solve ${{\bf{W}}_i}[{k_i}]$.

(8) $$li{m_{t \to \infty }}{e^{ - {{\bf{L}}_i}[{k_i}]t}} = {\bf{1}}{{\bf{W}}_i}[{k_i}],$$

where ${{\bf{L}}_i}[{k_i}]$ is the Laplacian matrix of the subdigraph composed by AUV- $i$ and its neighbors in the ${k_i}$ loop, ${{\bf{G}}_i}[{k_i}]$. ${{\bf{L}}_i}[{k_i}] = {{\bf{A}}_i}[{k_i}] - {\Delta _i}[{k_i}]$, ${{\bf{A}}_i}[{k_i}] = {[{a_{ij}}[{k_i}]]_{{N_i}[{k_i}] \times {N_i}[{k_i}]}}$ is the adjacency matrix, the elements $a_{ij}[k_i]=\left\{ {\matrix{(ord_{R_{j}}-ord_{R_{i}}) *q, if \;ord_{R_{i}} \lt ord_{R_{j}} \cr C, others \qquad \qquad \qquad \qquad \qquad \cr } } \right.,\Delta_i[k_i]=diag\{ D_i[k_i]\}\,{\rm is\; the\; degree \;matrix\;of\,{\bf{G}}_i}[k_i],$ ${D_i}[{k_i}] = \sum\nolimits_{j = 1}^{{N_i}[{k_i}]} {{a_{ij}}} [{k_i}]$, $q \ge 1$ and $C \ge 1$ are two parameters decided by the confidences of neighbors. An example is shown in Fig. 4, it is the sub-digraph ${{\bf{G}}_3}[{k_3}]$ of AUV- $3$ in the ${k_3}$ updating loop. The values of edges are ${a_{ij}}[{k_i}]$.

### Hybrid coordination strategy

TOLC is fast and simple, but it is invalid when the AUVs are hampered by obstacles. Meanwhile, ADWCC is good at accomplishing the tasks with distributed local information in complicated environments, but its time consumption is high due to searching neighbors loop by loop. Therefore, we combine both controllers to a new hybrid coordination strategy (Strategy 1).

$Strategy\;1$:

(1) Each AUV first uses TOLC to determine the pyramid distribution and to assign the positions in pyramid pattern to the AUVs to generate initial non-intercrossing trajectories, then each AUV moves along the initial non-intercrossing trajectory to its destination.

(2) When hampered by obstacles, the hampered AUVs switch to ADWCC to update trajectories loop by loop until they arrive at the destinations.

$Theorem\;1$: With Strategy 1, the AUVs can avoid obstacles and converge to the pyramid pattern fast.

Before the provement, we introduce several lemmas.

$Lemma\;3$ (Horn & Johnson, 2012): The necessary and sufficient condition for the convergence of a matrix ${\bf{A}}$ is that its spectral radius is less than 1, $\rho ({\bf{A}})\lt 1$, and the smaller the spectrum radius, the faster the convergence.

$Lemma \;4$ (Horn & Johnson, 2012): For a matrix ${\bf{A}} \in {\mathbb {C}^{n \times n}}$, its arbitraty norm is expressed by $||\cdot||$, then $\rho({\bf A})\leq ||{\bf{A}}||$.

${\bf{Proof}}$:

1. According to lemma 1, the covariant matrix and its eigenvalues and eigenvectors can present the topology of MSAS. Though the eigenvalues present the scales of the distribution, the scales of both distributions are fixed and not changeable in this study. Then we only compare the eigenvectors and the average positions.

In (5), ${{\bf{v}}_{pmax}} = {{\bf{v}}_{cmax}}$ and ${{\bf{o}}_p} = {1 \over N}\sum\nolimits_{i = 1}^N {{{\bf{p}}_{c{R_i}}}}$. The average positions and the principal components of positions are coincided. It means that the position and orientation of both distributions are the same. It shows that the pyramid distribution approximates the initial distribution.

2. With the proposed strategy, subgroups, $Sg_{ini}^i$ and $Sg_{py}^i$, with one or two positions are seperated, and $Sg_{ini}^i \cap Sg_{ini}^{i - 1} = \emptyset$, $Sg_{py}^i \cap Sg_{py}^{i - 1} = \emptyset$. Then combined-subgroups $Sg_{com}^i$ are obtained. Based on lemma 2, $\sum d is = min(({e_{11}} + {e_{22}}),({e_{12}} + {e_{21}}))$ in each $Sg_{com}^i$. As a result, arbitrary two edges are non-intercrossing. Then it satisfies (4).

3. Since the asynchronous discrete consensus algorithm is a discrete Markov chain process, the convergent condition of the asynchronous discrete consensus algorithm is that the each loop is convergent. Then it needs that the spectral radius of ${\bf{1}}{{\bf{W}}_i}[{k_i}]{{\bf{C}}_{i1}}[{k_i}]$ is less than 1, $\rho ({\bf{1}}{{\bf{W}}_i}[{k_i}]{{\bf{C}}_{i1}}[{k_i}])\lt 1$, according to lemma 3.

(1) According to (7), each element of ${{\bf{C}}_{i1}}[{k_i}]$ meets ${\beta _{ij}}[{k_i}] \in [0,0.5]$, shown in Eq. (4), then $\rho ({{\bf{C}}_{i1}}[{k_i}])\lt1$.

(2) Since the topologies are connected with Assumption 1, then Laplacian matrix ${{\bf{L}}_i}[{k_i}]$ is irreducible. The transition matrix of the discrete Markov chain is ${{\bf{P}}_i}[{k_i}] = {e^{ - {{\bf{L}}_i}[{k_i}]t}}$ (see (8)), then $\rho({e^{ - {{\bf{L}}_i}[{k_i}]t}})\leq || {e^{ - {{\bf{L}}_i}[{k_i}]t}}||$ according to lemma 4.

(3) Since $||{e^{ - {{\bf{L}}_i}[{k_i}]t}}|| \lt 1$, then we get that

$||{e^{ - {{\bf{L}}_i}[{k_i}]n}}|| \lt ||{e^{ - {{\bf{L}}_i}[{k_i}](n-1)}}||||{e^{ - {{\bf{L}}_i}[{k_i}]}}||\lt ||{e^{ - {{\bf{L}}_i}[{k_i}](n-1)}}||.$

Consequently, $||lim_{t \rightarrow \infty}e^{-L_{i}[k_i]t}||$ is the minimum norm. Then $\rho (li{m_{t \to \infty }}{e^{ - {{\bf{L}}_i}[{k_i}]t}})$ is the smallest spectral radium. As a result, the weight vector ${{\bf{W}}_i}[{k_i}]$ calculated by (8) makes the convergent speed of each loop fastest and improves the convergent speed of the MSAS according to lemma 3.

## Simulation results and discussion

**Figure 5:**
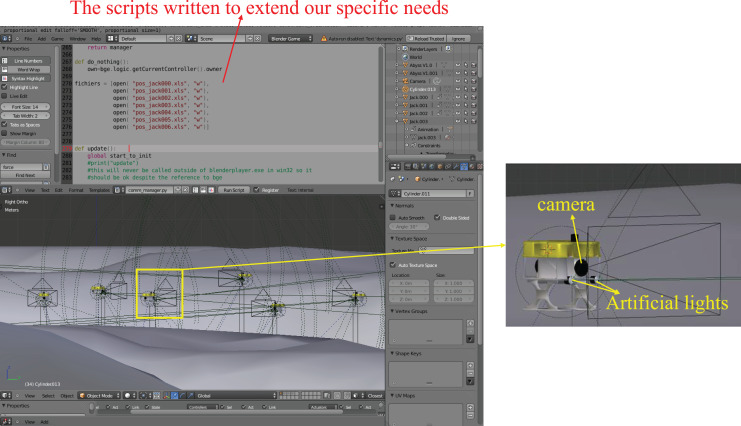
3D simulation environment constructed in Blender with seabed and fish.

To test the performance of the proposed hybrid coordination strategy, the simple 2D simulations in MATLAB and the 3D simulations with several AUV models structured based on the real CISCREA in Blender (see Fig. 5) have been done.

### The pyramid building in MATLAB

**Figure 6:**
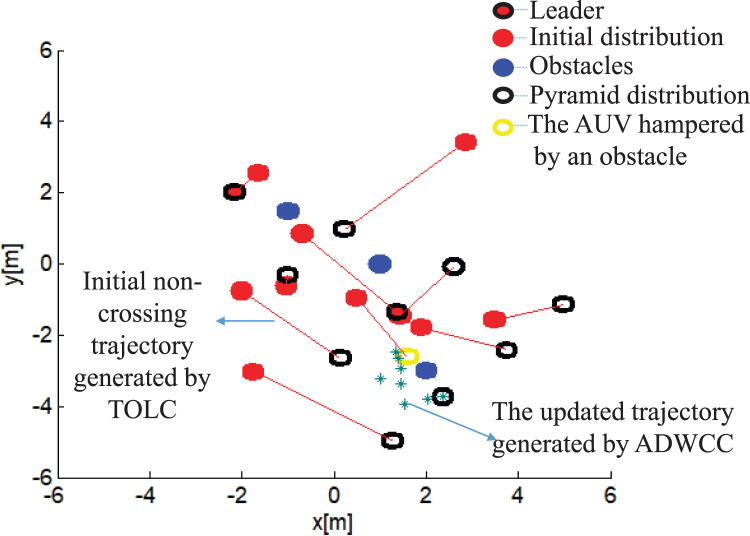
An example of pyramid building with obstacles.

In MATLAB, a red point is used to express the initial position of an AUV, while a blue point is used to express an obstacle. The expected relative distance and angle between each child and its parent are set to the lower limit values: ${d_e} = 2.5\;m$ and ${\varphi _e} = {20^\circ }$ when taking the short working range of underwater cameras into account. Noises are randomly added to simulate the image processing errors ${\delta _{img}}$ and movement errors ${\delta _{move}}$, both ${\delta _{img}}$ and ${\delta _{move}}$ are within $\pm 10\%$ of ${d_e}$ and ${\varphi _e}$. Figure 6 shows an example of trajectories of AUVs to build a pyramid pattern in the obstacle environment. The black rings are the destinations of AUVs in the pyramid pattern, in which the black ring including a red point is the leader’s position. The red lines are the edges connecting the assignment results in both distributions, they are also the trajectories of AUVs without hampered by obstacles. The yellow ring is the position stopped by obstacles, while the green star dashed curve is the updated trajectory of this AUV with the ADWCC.

**Figure 7:**
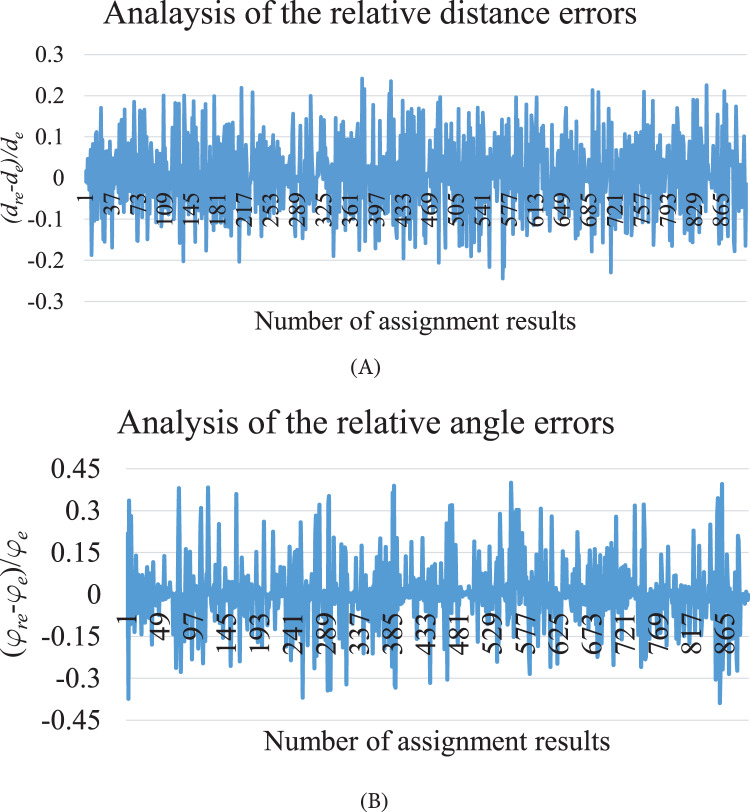
The relative distance errors and relative angle errors between each child and its parent at the convergent state. (A) The relative distance errors. (B) The relative angle errors.

To test the stability, 100 times simulations have been done by 10 AUVs with different initial distributions and obstacles and with the noises ${\delta _{img}}$ and ${\delta _{move}}$ in MATLAB. To analyze the errors of the converged pyramid pattern, the ratios $({d_{re}} - {d_e})/{d_e}$ and $({\varphi _{re}} - {\varphi _e})/{\varphi _e}$ are computed and shown in Fig. 7, where ${d_{re}}$ and ${\varphi _{re}}$ are the convergent relative distance and relative angle between each AUV and its parent.

Figure 6 shows that the built formation is within the initial distribution range, the initial trajectories are non-intercrossing with short distances; the hampered AUVs arrive at their destinations by updating trajectories. Figure 7 presents that the relative distance errors are all within 25% of ${d_e}$, while the relative angle errors are all within 40% of ${\varphi _e}$. The errors around 40% of ${\varphi _e}$ are generated due to the large threshold errors of ADWCC, and the angle errors are more sensitive due to the small value of ${\varphi _e}$. Both Figs. 6 and 7 illustrate that the proposed hybrid coordination strategy can collect all the AUVs together to build the pyramid pattern successfully.

### The pyramid building in Blender

**Figure 8:**
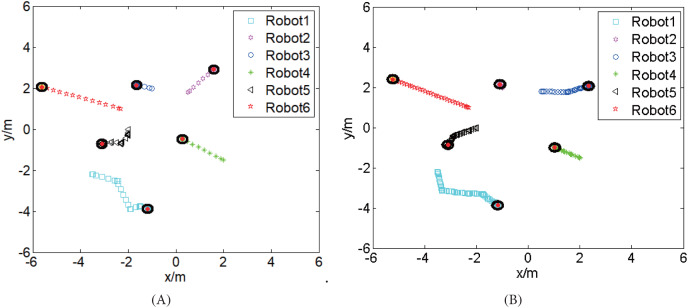
The trajectories of six CISCREAs to build the pyramid from the same initial distribution. (A) With the proposed hybrid coordination strategy. (B) With the discrete consensus algorithm.

To further test the performance of the proposed strategy, the CISCREA model (see Fig. 5) is built based on the real CISCREA and its hydrodynamic model (Yang et al., 2015). In the 3D simulation environment, each CISCREA perceives the information by the on-board cameras and compasses. We set ${d_e} = 3.5\;m$, ${\varphi _e} = {30^\circ }$ according to the parameters of the equipped cameras. Then each CISCREA plans and/or updates its trajectories with the proposed strategy. Here we present an situation that CISCREA-1 and CISCREA-5 are hampered at the beginning. In this case, we achieve the pyramid building, the AUVs’ trajectories are displayed in Fig. 8A. To compare with the ADWCC, we also did the simulations based on the discrete consensus algorithm with the same initial distribution, and the EPR is also obtained using TOLC. The trajectories are dispicted in Fig. 8B. The time consumptions of both methods are about 8 and 13 min, respectively. We repeated the pyramid building many times with both methods, and the time consumptions, ${t_{cvg}}$, are recorded in Table 1. The minimum time-cost of the proposed hybrid coordination, 2 min, happens when no CISCREAs are blocked by obstacles.

**Table 1:** Comparation of both methods to build the pyramid pattern.

	The proposed hybrid coordination	The discrete consensus algorithm
$max \left\{ {{|{d_{r{e_i}}} -\; {d_e}|} \over {{d_e}}}|i \in \{ 1, \ldots ,N\} \right\}$	25.14%	40.04%
$max \left\{ {{|{\varphi _{r{e_i}}} -\; {\varphi _e}|} \over {{\varphi _e}}}|i \in \{ 1, \ldots ,N\} \right\}$	39.98%	46.96%
${t_{cvg}}$	2–10 min	12–20 min

From Fig. 8, we find that both the proposed hybrid strategy and discrete consensus algorithm can achieve the formation building, and their trajectories are non-intercrossing. Table 1 illustrates that the proposed hybrid method has high accuracy and low time consumption. The reasons includes that (1) the relative errors of CISCREAs moving along the initial linear trajectories are smaller, because there are no accumulation of errors caused by repeated loops; (2) the weights of ADWCC are calculated by introducing the limited transition matrix; (3) the hampered CISCREAs need less updating loops to arrive at their destinations with corrected relative information, because most of their neighbors have already arrived at their destinations.

## Discussion

The hybrid coordination strategy makes the dis-ordered AUVs fast converge to the pyramid pattern without the collision among AUVs or the collision with obstacles. The simulations present good results. Although it is only a sub-optimal result, the relative distance error is small, and the relative angle error is large but sufficient for practical applications. The time consumption is much smaller than that of discrete consensus algorithm.

The TOLC determines the pyramid formation, whose distribution is symmetrical within the area of the initial distribution. Meanwhile, TOLC achieves the positions of assignment in both distributions and generates the non-intercrossing straight trajectories. The ADWCC realizes the trajectories updating when hampered by obstacles. Both parts take the fast convergence into account.

However, the accuracy of the hybrid coordination strategy can be enhanced further with increasing the time cost, especially the relative angle between each AUV and its parent. But the current accuracy is sufficient to meet application requirements, since the relative distances and angles will be continuously fine-tuned when moving forward. As a result, the proposed hybrid coordination strategy saves the convergence time of MSRSs and enhances the practical value of the system.

## Conclusion

We present a hybrid coordination strategy to build a planar pyramid pattern fast. It includes two parts: TOLC and ADWCC. TOLC is an efficient sub-optimal method, by combining of geometric way and algebraic way, to tend the pyramid distribution to the initial distribution and to match up the positions in both initial and pyramid distributions to get a short initial trajectory. ADWCC is a distributed method to steer the hampered AUVs arriving at their destination with the neighbors information. And ADWCC employs the limited transition matrix to calculate the weights for accelerating the convergent speed of each updating loop. A theoretical proof is reported to analyze the rapidity. The simulations in both MATLAB and Blender have also verified the feasibility, stability, and rapidity of the proposed method. The comparison with the discrete consensus algorithm also provides that the proposed strategy is more accurate with lower time consumption.

In the future, the random link failures of topologies due to the limited perception ability of on-board camera will be considered. In addition, formation maintenance and formation re-building will be further studied to make the MSRSs capable of achieving the given tasks.

## Supplemental Information

10.7717/peerj-cs.1358/supp-1 Supplemental Information 1 A video of formation building with the hybrid coordination strategy based on on-board cameras. The video corresponds to the trajectories presented in Fig. 8A.

10.7717/peerj-cs.1358/supp-2 Supplemental Information 2 A video presents the formation building with the consensus algorithm based on on-board cameras. The video corresponds to the trajectories presented in Fig. 8B.

10.7717/peerj-cs.1358/supp-3 Supplemental Information 3 Code of the simulation of the hybrid coordination. “example_0~6” are the main codes for each Ciscrea robot modeled in Blender.

10.7717/peerj-cs.1358/supp-4 Supplemental Information 4 Code of the simulation of the consensus algorithm. “example_0~6” are the main codes for each Ciscrea robot modeled in Blender.

## Additional Information and Declarations

### Data Availability

The following information was supplied regarding data availability: The simulations in Blender and codes are available in the Supplemental Files.
